# Safety and Efficacy of TKIs in very Elderly Patients (≥75 Years) with Chronic Myeloid Leukemia

**DOI:** 10.3390/jcm13010273

**Published:** 2024-01-03

**Authors:** Alessandro Costa, Elisabetta Abruzzese, Roberto Latagliata, Olga Mulas, Ida Carmosino, Emilia Scalzulli, Maria Laura Bisegna, Claudia Ielo, Maurizio Martelli, Giovanni Caocci, Massimo Breccia

**Affiliations:** 1 1Hematology Unit, Businco Hospital, Department of Medical Sciences and Public Health, University of Cagliari, 09121 Cagliari, Italy; alessandrocosta161195@gmail.com (A.C.); mulasolga@unica.it (O.M.); giovanni.caocci@unica.it (G.C.); 2 Hematology Unit, S. Eugenio Hospital, ASL Roma 2, Tor Vergata University, 00144 Rome, Italy; 3 Hematology Unit, Belcolle Hospital, 01100 Viterbo, Italy; 4 Hematology, Department of Translational and Precision Medicine, Az. Policlinico Umberto I-Sapienza University, 00185 Rome, Italy

**Keywords:** chronic myeloid leukemia, tyrosine kinase inhibitor, very elderly, safety, efficacy, outcome, dose optimization

## Abstract

Background: While the outcomes of chronic phase chronic myeloid leukemia (CP-CML) patients aged over 65 years have been extensively evaluated in real-life experiences, limited data exist for the very elderly population (i.e., aged ≥ 75 years), especially for next-generation tyrosine kinase inhibitors (TKIs). In this retrospective study, we sought to evaluate the safety and efficacy of TKIs in this particular setting of patients. Methods: We conducted a retrospective analysis of a multicenter cohort of 123 newly diagnosed CP-CML very elderly patients. Results: The median age at diagnosis was 80 years (range: 75–96). In the first line, 86.1% of patients received imatinib, 7.1% dasatinib, 5.6% nilotinib, and 0.81% received bosutinib. A total of 31 patients (25.2%) switched to second-line therapy, nine patients to a third line, and one patient to a fourth line of therapy. Resistance to treatment was the primary reason for switching therapy in both the first (64.5%) and second lines (77.7%). At diagnosis, reduced doses were administered in 36.5% of patients, in 61.2% in the second line, and in all patients in subsequent lines of therapy. In the first-line setting, 71.9% of patients achieved an early molecular response (EMR, i.e., 3-month *BCR::ABL1*^IS^ < 10%); at 6, 12, and 24 months, MR3 was reached by 35.7%, 55.7%, and 75.0% of patients, respectively, with 16.6%, 35.7%, and 51.7% achieving a deep molecular response (DMR) at the same time points. Treatment-free remission (TFR) was successfully attempted in 11 patients. During the follow-up period, adverse events (AEs) were observed in 78.8% of patients, including 22 cases of cardiovascular AEs. Toxicity grade ≥ 3 was more commonly observed in patients treated with standard doses of TKIs compared to reduced doses (*p* = 0.033). Overall, the median follow-up was 46.62 months (range: 1.8–206.2), and 43 patients died due to non-CML-related causes. Three patients died due to disease progression to advanced (*n* = 1) and blastic (*n* = 2) phases. The 5-year overall survival (OS) for the entire cohort was 71.9% (95% CI: 0.63–0.81), with no significant difference between the patients treated with standard doses of TKIs compared to those treated with reduced doses (*p* = 0.35). Conclusions: TKIs appear to be safe and effective even in very elderly CML patients, and dose optimization strategies yield satisfactory molecular responses for adequate disease control with an improved safety profile.

## 1. Introduction

In chronic myeloid leukemia (CML), age as a prognostic factor has been significantly recognized and has been included in the Sokal and EURO scores [1,2]. However, the advent of tyrosine kinase inhibitors (TKIs) has revolutionized the clinical strategy for the disease, substantially enhancing the outcomes, even among older patients [3]. In the pre-TKI era, the treatment approach for elderly CML patients primarily focused on minimizing the disease burden, considering therapy-related toxicities and the frailty of the patients [4].

Significantly, starting from 2018, the average life expectancy for male CML patients aged over 65 is approximately 16 years, while for those aged over 75, it is around 9 years [5]. However, despite these encouraging statistics, age continues to be considered in the latest ELTS score, albeit with reduced influence on the outcomes [6]. Managing elderly CML patients continues to pose a challenge, highlighting the need for tailored approaches in this population.

Comorbidity, geriatric syndromes, and polypharmacy are frequently heightened in elderly CML patients [7]. Furthermore, age-related variations in pharmacokinetic and pharmacodynamic parameters can alter drug tolerance and efficacy [8]. Consequently, older patients are often excluded from participation in clinical trials, and the available data primarily stem from real-world experiences [9]. Notably, Rohrbacher et al. demonstrated that CML patients enrolled in clinical trials are, on average, ten years younger than those who are not included [10]. While imatinib (IMA) has demonstrated excellent safety and efficacy, even in very elderly patients (i.e., aged ≥ 75 years) [11], there is limited knowledge regarding the use of next-generation TKIs in this population. Although age does not hinder the achievement of molecular responses in older patients treated with second (2G) or third-generation (3G) TKIs, the presence of comorbidities may limit their prescription [12]. Consequently, a careful evaluation is necessary to mitigate the risk of life-threatening adverse events (AEs).

Dose optimization strategies have shown favorable outcomes in the real world in minimizing toxicity while ensuring the attainment and sustenance of molecular responses [13,14,15,16]. This approach can be particularly advantageous for older or frail patients at diagnosis or during follow-up, helping to mitigate persistent or low-grade AEs, such as fatigue or edema associated with IMA and diarrhea linked to bosutinib (BOS) [17]. Furthermore, the goal of limiting cardiovascular and pulmonary toxicities associated with nilotinib (NIL), dasatinib (DAS), and ponatinib (PON) can be accomplished through dose reduction in patients achieving an optimal response [12].

Limited research has specifically focused on the efficacy and safety of TKIs in very elderly CML patients. Furthermore, data on the feasibility of using PON and asciminib (ASC) in very elderly patients who have shown resistance to prior treatment lines are still limited. Therefore, the aim of this retrospective multicentric study was to evaluate the safety and efficacy of TKIs in a cohort of 123 newly diagnosed very elderly CML patients, providing valuable real-world data.

## 2. Materials and Methods

We performed a retrospective analysis on 123 newly diagnosed CML patients aged ≥ 75 years from January 2003 to January 2023. Four Italian centers collaborated and provided the required data after obtaining informed consent from all the patients. The inclusion criteria were as follows:

- Confirmed diagnosis of CP-CML
- Age at diagnosis ≥ 75 years old
- Frontline treatment with TKIs

The diagnostic and response criteria were based on current European LeukemiaNet (ELN) recommendations [18]. The information extracted from patients’ records included data about their age, medical history, medication history, disease stage, and risk of progression based on the Sokal and ELTS scores at the time of diagnosis [1,6]. An approach of a complete case analysis was adopted, reporting only data from patients with complete information. The comorbidities burden was evaluated using the Charlson Comorbidity Index (CCI) [19]. Data regarding the treatment duration for each TKI in the first, second, third, and fourth lines of therapy were collected, including information on the initial doses and any dose reductions during follow-up. Hematological and non-hematological toxicities were assessed and graded based on the Common Terminology Criteria for Adverse Events (CTCAE) version 5.0 [20].

Bosutinib was administered as a first-line treatment in a patient enrolled in the BFORE registration trial (NCT02130557), which compared bosutinib with imatinib as a first-line therapy [21].

The cytogenetic responses were evaluated using standard G-banded karyotype on bone marrow (BM) aspirates or by fluorescent *in situ* hybridization (FISH) on BM interphasic cells. The transcription levels of *BCR::ABL1* were evaluated using RQ-PCR in certified laboratories, and the molecular responses (MR) were defined according to standardized criteria as a Major Molecular Response (MMR) (*BCR::ABL1*^IS^ ≤ 0.1%) and a Deep Molecular Response (DMR) (MR4.0, *BCR::ABL1*^IS^ ≤ 0.01%; MR4.5, *BCR::ABL1*^IS^ ≤ 0.0032%; MR5.0, *BCR::ABL1*^IS^ ≤ 0.001%) [18].

The continuous variables were reported as medians and ranges, while the categorical variables were presented as frequencies and percentages. The overall survival (OS) was calculated from the date of diagnosis until the time of death or last follow-up, while the event-free survival (EFS) was calculated from the initiation of each TKI treatment until treatment failure, discontinuation for any reason, or progression to the accelerated phase (AP) or the blast phase (BP). The progression-free survival (PFS) was calculated from the start date until progression to the AP or BP. Group comparisons were conducted using appropriate statistical tests, such as unpaired *t*-tests, chi-square tests, and Fisher’s exact test. A significance level of *p* < 0.05 was considered statistically significant. Survival analysis was performed using Kaplan–Meier curves, and the differences were assessed using log-rank tests. Univariate and multivariate analyses were carried out using Cox regression analysis to determine the hazard ratios (HR) and 95% confidence intervals (95% CI) for the factors associated with survival. The analyses were conducted using R (R Core Team 2020, Vienna, Austria) and RStudio (RStudio Team, 2020, Boston, MA, USA).

## 3. Results

### 3.1. Patients

A total of 123 very elderly newly diagnosed CML patients were included in the study. The patients’ characteristics are displayed in Table 1. The median age at diagnosis was 80 years (range: 75–96). Among the patients, 48% were aged 85 years or older. All the patients were in the chronic phase (CP) of CML. Based on the Sokal score, 0.8% of the patients had a low risk, 71.5% had an intermediate risk, and 27.6% had a high risk. According to the ELTS score, 3.2% of the patients were classified as low risk, 61.7% as intermediate risk, and 34.9% as high risk. Baseline comorbidities were documented in 118 out of 123 patients (95.9%).

Collectively, 103 patients (83.7%) had cardiovascular (CV) risk factors at baseline, including hypertension, dyslipidemia, type 2 diabetes, and a smoking habit, while 52 (42.2%) had a history of cardiovascular disease (CVD). Among these patients, 7 (5.6%) had chronic heart failure and 13 (10.4%) had atrial fibrillation. Moreover, 10 patients (8.1%) had a history of myocardial infarction and 13 (10.4%) of stroke or transient ischemic attack (TIA). The CCI was 0 in 55 patients, 1 in 28 patients, and ≥2 in 40 patients. In total, 116 patients (94.3%) assumed concomitant medications for any causes, with an average of 4.5 drugs per person (range: 0–13). Polypharmacy, defined as the use of ≥5 drugs, was found in 47% of the patients.

### 3.2. Response Rate and Survival

In the frontline therapy, the patients were treated with IMA (*n* = 101; 86.1%), DAS (*n* = 9; 7.1%), NIL (*n* = 7; 5.6%), and BOS (*n* = 1; 0.81%). The median doses and duration for each line of therapy are shown in Table 2.

Dose-reduced TKIs were administered in 45 patients (36.5%) in the first line; among them, 40 patients received dose-reduced IMA, predominantly at 300 mg/day (range: 200–300), while 4 patients were administered with DAS at 50 mg/day, and 1 patient received NIL at 300 mg/day. Dose reduction was performed at diagnosis in 30 patients (IMA, *n* = 27; DAS, *n* = 3) and in 15 patients (IMA, *n* = 13, DAS, *n* = 1, NIL, *n* = 1) due to intolerance after a median follow-up time of 7.78 months (range: 0.36–85.34). Once reduced, the reduction was maintained for a median time of 31.02 months (range: 0.49–129.1) until the last follow-up or a switch of therapy. The patients treated with reduced doses of TKIs (RD-TKIs) were older compared to those treated with standard doses (SD-TKIs) (median age: 79 years vs. 82 years, *p* = 0.0003), with no significant differences in the number of comorbidities between the two groups (*p* = 0.11).

Overall, the 3-month cytogenetic response was assessed in 75 patients, with 29.3% achieving a Partial Cytogenetic Response (PCyR) and 45.3% achieving a Complete Cytogenetic Response (CCyR). Among the patients evaluable for MR, 71.9% (64/89) achieved an early molecular response (EMR, i.e., 3-month *BCR::ABL1*^IS^ < 10%). At 6, 12, and 24 months of treatment, MR3 was achieved by 35.7% (30/84), 55.7% (39/70), and 75.0% (42/56) of patients, respectively. Additionally, at these time points, 16.6%, 35.7%, and 51.7% of the patients achieved a DMR. Overall, 29.9% of the patients did not achieve an MR in the first-line treatment.

The cumulative incidence of MMR at 6, 12, and 24 months in the evaluable patients was 37% (95% CI: 26–48), 56% (95% CI: 43–67), and 81% (95% CI: 67–89), respectively, for the patients treated with SD-TKIs. For those treated with RD-TKIs, the cumulative incidence was 18% (95% CI: 6.9–32), 52% (95% CI: 32–68), and 74% (95% CI: 51–87). No statistically significant differences were observed between the two patient groups (*p* = 0.3).

**Figure 1:**
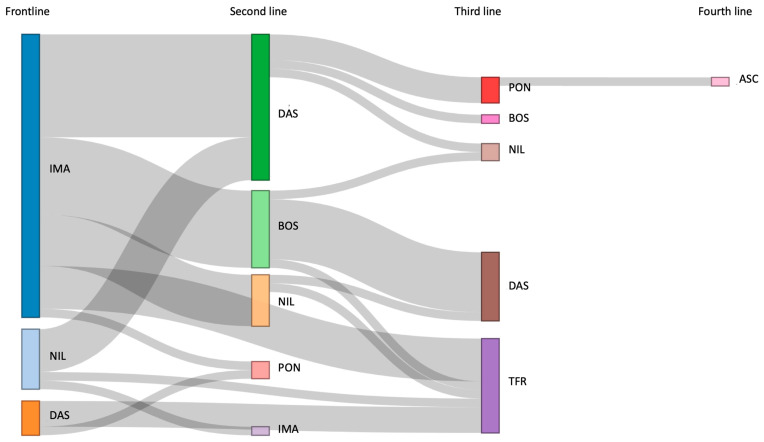
Treatment pathways across lines of therapy. ASC, asciminib; BOS, bosutinib, DAS, dasatinib; IMA, imatinib; NIL, nilotinib; PON, ponatinib; TFR, treatment-free remission.

Thirty-one patients (25.2%) switched to a second line of treatment (Figure 1), mainly for resistance to treatment (64.5%): thirteen patients switched to DAS (41.9%), nine to BOS (29.0%), six to NIL (19.3%), two to PON (6.4%), and one to IMA (3.2%).

In the second line, 19 patients (61.2%) were treated with RD-TKIs: 8 patients received BOS, 5 received NIL, 5 received DAS, and 1 patient received PON. Specifically, BOS was predominantly administered at 100 mg/day (range: 100–400), NIL at 600 mg/day (range: 400–600), DAS at 50 mg/day (range: 50–80), and PON at 15 mg/day. In 7 patients (BOS, *n* = 2; DAS, *n* = 2; NIL, *n* = 2; PON, *n* = 1), the TKIs were started at a reduced dosage, while in another 12 (BOS, *n* = 6; DAS, *n* = 3; NIL, *n* = 3), a reduction became necessary during follow-up due to intolerance after a median time of 10.61 months (range: 1.02–41.8). The dose reduction was sustained in all the patients for a median time of 36.05 months (range: 3.87–116.3), until the last follow-up or a switch of therapy.

Over the 24 months of follow-up, 48.3% of the patients achieved either MR3 or DMR, with only one patient being unevaluable for MR. In the second line as well, the cumulative incidence of MMR at 6, 12, and 24 months did not differ between those administered with SD-TKIs or RD-TKIs (*p* = 0.2).

Nine patients switched to a third line of treatment, primarily due to resistance in seven patients (77.7%) and intolerance in two patients (22.2%). Mutation analysis identified the T315I mutation in one patient and the G442E mutation in another. PON was administered in five patients, while two patients switched to NIL and two to DAS and BOS each. Four patients achieved an MR3 or DMR during the 24 months of follow-up, the other four patients never achieved any MR, while one patient was not evaluable for MR.

Only one patient switched to a fourth line due to the T315I mutation and initiated ASC at a dose of 200 mg BID. The basal *BCR::ABL1*^IS^ was 35%; after 3 months of treatment, the patient achieved the MMR and maintained it until the last follow-up. After 58 days of treatment, the patient experienced a grade 3 increase in transaminases, leading to a temporary discontinuation of ASC. The TKIs were reintroduced at a reduced dosage once the toxicity resolved, and no new episodes were reported thereafter.

Overall, the median follow-up was 46.62 months (range: 1.8–206.2). Out of the total cohort, 43 patients (34.9%) died, mainly from causes unrelated to CML (93%), while 3 patients died due to progression to BP.

**Figure 2:**
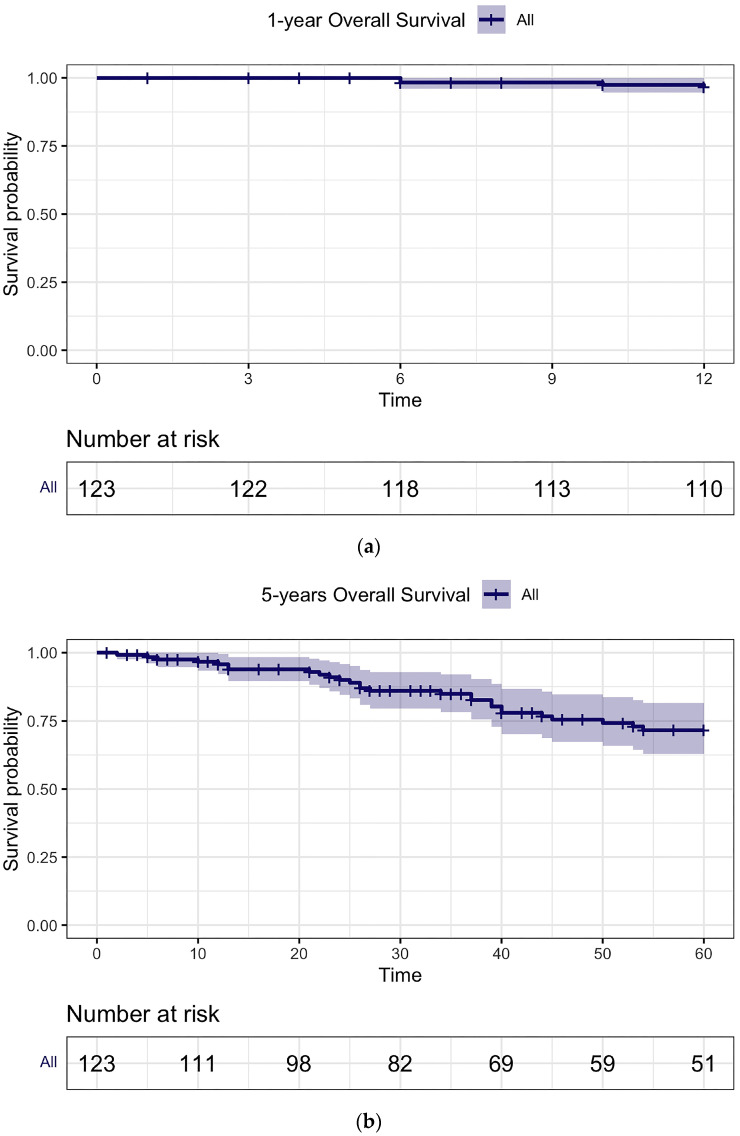

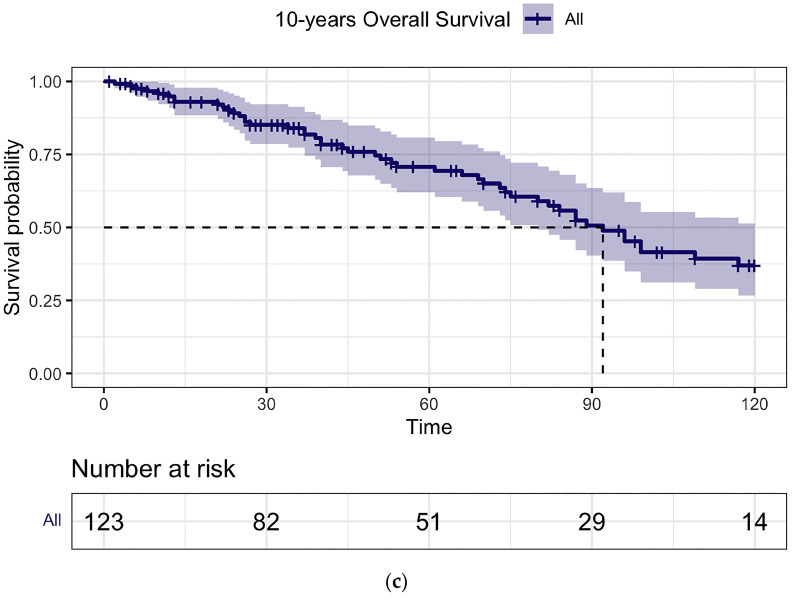
Overall Survival, respectively, at (**a**) 1 year, (**b**) 5 years, and (**c**) 10 years of follow-up.

The OS rates for the entire cohort were 96.6% (95% CI: 0.93–0.99) at 1 year, 71.9% (95% CI: 0.63–0.81) at 5 years, and 37.7% (95% CI: 0.27–0.52) at 10 years of follow-up (Figure 2).

**Figure 3:**
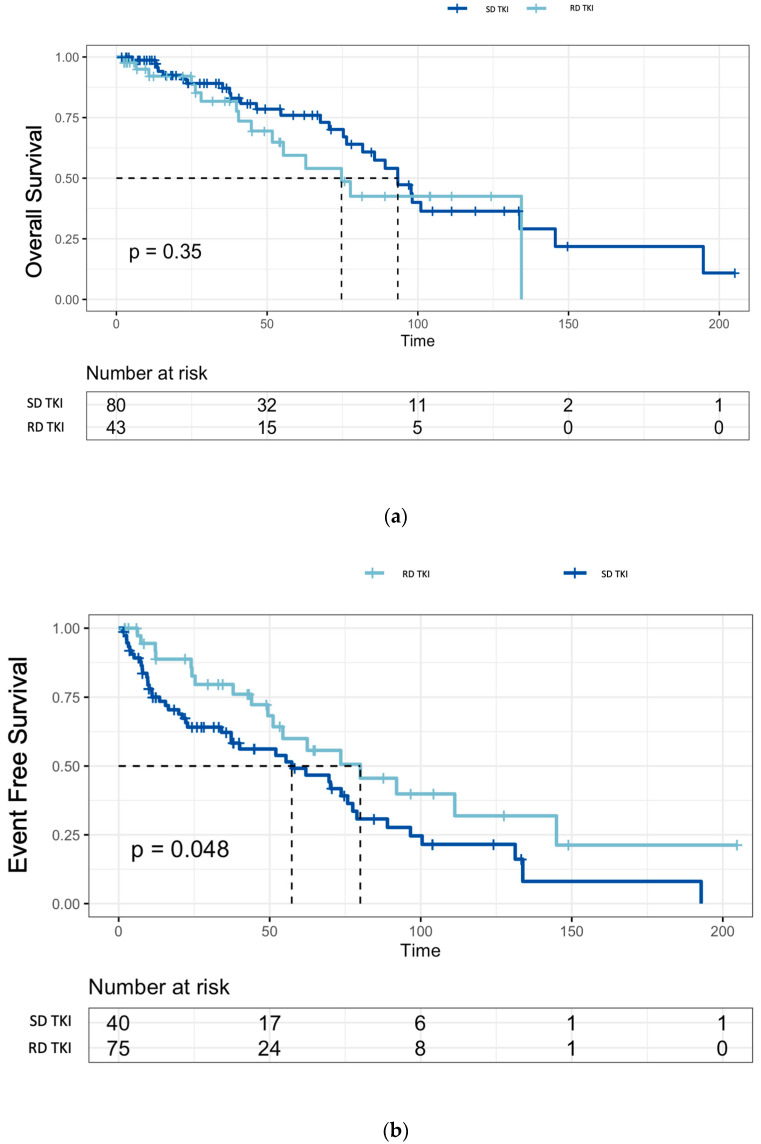
(**a**) Overall Survival and (**b**) Event-Free Survival in the Standard-Dose TKIs (SD-TKIs) and Reduced-Dose TKIs (RD-TKIs) cohorts.

There was no significant difference in the OS between the patients treated with SD-TKIs or RD-TKIs (*p* = 0.35) (Figure 3a). In the evaluable patients (*n* = 115), the EFS was 62 months (95% CI: 44–78) in the first-line treatment, while it was 57 months (95% CI: 20–90) in the second-line treatment. Additionally, the median EFS of the patients treated with the SD-TKIs was significantly shorter compared to the RD-TKIs (80 months vs. 57 months, *p* = 0.048) (Figure 3b).

Overall, the 5-year PFS was 95.3% (95% CI: 0.90–1). Univariate and multivariate analyses were performed to assess the predictive role of various baseline characteristics (age at diagnosis, sex, Sokal score, ELTS score, CCI, comorbidities, and polypharmacy) as well as treatment-related factors (number of treatment lines, grade ≥ 3 AEs) on the survival outcomes in the entire patient cohort. Among these factors, only age at diagnosis ≥80 years demonstrated a negative prognostic impact in both the univariate analysis (*p* = 0.00017) and the multivariate analysis (*p* = 0.00079), indicating that a very old age at diagnosis was associated with a poor survival outcome.

### 3.3. Adverse Events

**Figure 4:**
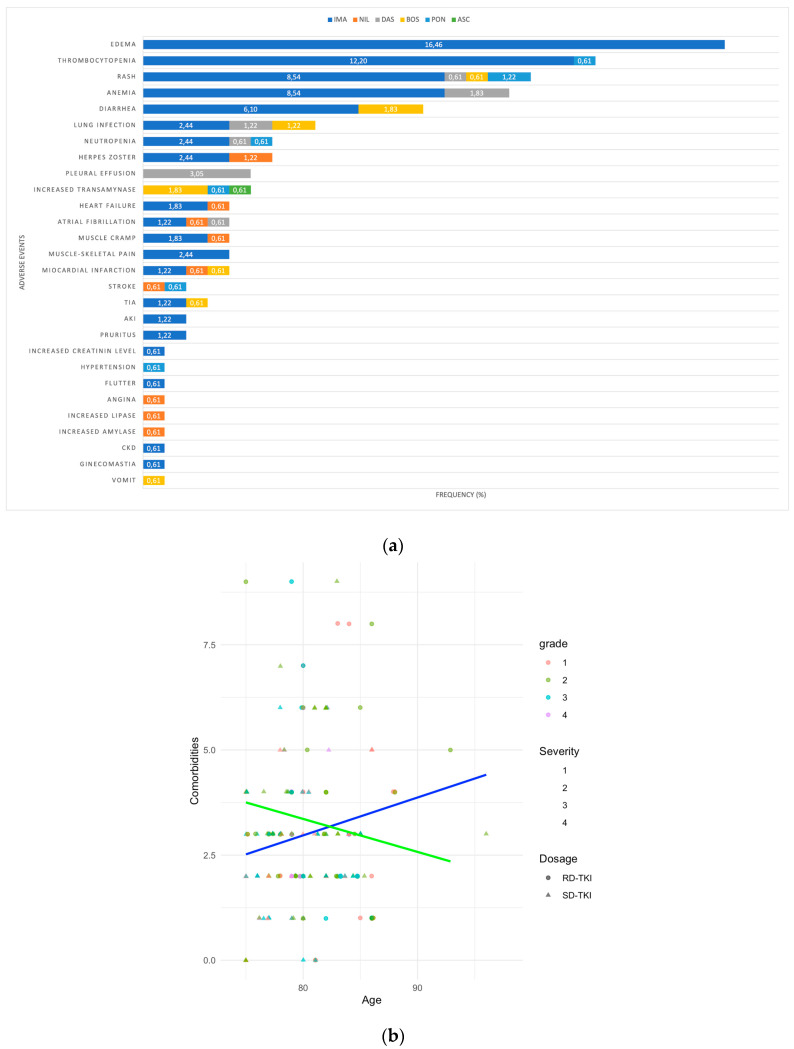
(**a**) Frequency of adverse events (AEs) by drug: bar chart illustrating frequency of AEs categorized by drug in the cohort. (**b**) Association of AEs with age and comorbidities: scatter plot depicting the relationship between age (years) and number of comorbidities with regression lines for Standard dose-TKIs (blue line) and Reduced dose-TKIs (green line). AKI, Acute Kidney Injury; CKD, Chronic Kidney Disease; TIA, Transient Ischemic Attack.

During the follow-up period, AEs were observed in 97 patients (78.8%), with the majority classified as non-hematological (72.4%) and graded as 1–2 according to the CTCAE criteria. Figure 4a provides a summary of the most commonly reported AEs. The most frequently observed AEs included edema, diarrhea, and cutaneous rash, predominantly of grade 1 (range: 1–3).

Pleural effusion was detected in the patients treated with DAS both in the frontline (*n* = 1, 80 mg/day; *n* = 2, 100 mg/day) and second-line settings (*n* = 2, 100 mg/day). The median age of these patients was 79.3 years (range: 76–83), and the median time of onset was 34.6 months (range: 4.9–86.7) in the frontline setting and 11 months (range: 5–17) in the second-line setting. Permanent discontinuation of treatment was required for two patients, one with grade 3 and another with recurrent grade 2 pleural effusion, while the others were managed through dose reduction and/or specific therapy.

Overall, there were 22 cardiovascular adverse events (CVAEs) in 17 patients (13.8%), including 15 patients with CV risk or a CVD at baseline. Among these, 9 patients were <80 years old, while 6 patients were 80 years old or older. Specifically, in the first-line setting, 3 patients treated with IMA experienced acute heart failure, 3 had a myocardial infarction, 3 had a TIA, 3 had atrial fibrillation, and 1 had an atrial flutter. The average dose of IMA administered was 400 mg/day. Among the patients treated with next-generation TKIs as a first-line therapy, one patient receiving NIL at a daily dose of 600 mg experienced episodes of angina. In the second-line treatment, one patient receiving NIL at 600 mg/day exhibited acute heart failure and atrial fibrillation, while another patient suffered a stroke. A patient treated with DAS at 50 mg/day developed atrial fibrillation. A patient treated with BOS at 500 mg/day had a TIA, and newly diagnosed hypertension was observed in a patient treated with PON at a daily dose of 45 mg. In the third-line setting, a patient receiving PON at 15 mg/day experienced a stroke, and a patient treated with BOS at 500 mg/day had a myocardial infarction. No statistically significant difference was found between the SD-TKIs and RD-TKIs (*p* = 1).

Hematological AEs were observed across all treatment lines, typically manifesting with a median onset time of 3.53 months and mostly of grade 3 (range: 1–4). Among these, grade 3 thrombocytopenia (range: 1–4) was the most commonly observed hematological AE. Permanent discontinuation was necessary for three patients due to grade 4 hematological AEs, while most of the grade 1–3 AEs were managed through dose reductions or temporary drug withdrawal.

Collectively, grade *≥* 3 AEs occurred in 46 patients, with 35 treated with SD-TKIs and 11 with RD-TKIs (*p* = 0.033). No statistically significant differences were found in the OS between those who experienced grade *≥* 3 AEs and those who did not (*p* = 0.24). Discontinuation of the first-line treatment was required in 50 patients (40.7%), with 70% being temporary and 30% permanent. These interruptions were more frequent in patients aged ≥80 years (50%) compared to patients aged <80 years (30.5%, *p* = 0.042).

### 3.4. Treatment-Free Remission

Overall, treatment-free remission (TFR) was successfully achieved in 11 patients after a median follow-up of 81.80 months (range: 12.6–103.3) (Figure 1). Among these patients, 8 were administered frontline IMA (200 mg, *n* = 2; 300 mg, *n* = 3; 400 mg, *n* = 3), 2 received DAS (50 mg, *n* = 1; 80 mg, *n* = 2), and 1 received NIL (600 mg/day). Additionally, 2 patients underwent multiple lines of treatment: one, initially treated with IMA 400 mg/day, transitioned to NIL 600 mg/day, while another, initially receiving frontline IMA 300 mg/day, switched to BOS 200 mg/day. Specifically, discontinuation was considered in 7 patients based on the duration and depth of the MR according to ELN 2020 [18], while in 4 patients, it was performed due to the emergence of severe AEs. The median age at suspension was 85 years (range: 82–93). Furthermore, 5 patients were treated with first-line RD-TKIs (IMA 200 mg/day, *n* = 2; IMA 300 mg/day, *n* = 1; DAS, 50 mg/day, *n* = 1; DAS, 80 mg, *n* = 1). After a median follow-up of 15.9 months (range: 1–46), 8 patients maintained DMR, while molecular recurrence (MMR or higher) was observed in 3 patients (27.2%) after a median time of 10.6 months (range: 1–27). Consequently, the previous TKIs were restarted, and one out of the three patients regained a DMR (MR4).

## 4. Discussion

Improved life expectancy has led to more CML diagnoses in advanced age, with around 30% of patients falling into this group [22]. Enhanced survival rates in CML, comparable to the general population, mean that more patients age during treatment, complicating the decision-making process. This underscores the importance of evaluating the safety and efficacy of TKIs for very elderly patients.

Consistent with literature trends, most very elderly patients in our cohort received IMA [9]. However, there were no significant differences in the age (*p* = 0.32) or comorbidity count (*p* = 0.73) between those treated with IMA vs. 2G TKIs. Additionally, cardiovascular risk (CVR) factors or a history of CVD did not significantly impact the initial treatment choice, with no statistically significant difference between the IMA and next-generation TKI-treated patients (*p* = 0.77).

In recent decades, the ageing population has seen a rise in multimorbidity (i.e., two or more chronic conditions) and polypharmacy [23]. Despite this trend, evidence in the literature supports the efficacy of TKIs in this context, including in those aged ≥75 years [11,24,25,26]. Consistent with this, polypharmacy did not impact the patient outcomes in our study. Furthermore, while comorbidities at diagnosis have been linked to poorer outcomes [27], they did not significantly influence the survival in our cohort.

In the literature, experiences have been reported with symptoms ranging from moderate to severe in up to one-third of CML patients, while persistent mild symptoms were noted in up to 90% of cases [28,29]. Our cohort exhibited good tolerability to TKIs, and the development of grade ≥3 AEs did not significantly impact the patients’ prognoses. Importantly, pleural effusion, a known event during treatment with DAS [30,31,32], was reported in 22.7% of the patients treated with DAS in the first and second line, predominantly of mild grade and managed with specific therapy and temporary withdrawal of TKIs.

In addition, we observed CVAEs in 13.8% of the enrolled patients. Age is considered the primary driver for CV diseases [33,34], and our analysis shows, for the first time, the actual incidence of CVAEs in a large cohort of very elderly patients treated with various TKIs in subsequent lines of treatment. Interestingly, we observed a higher number of CVAEs in patients under 80 years old. One possible explanation, as mentioned earlier, could be a higher tendency to use SD-TKIs in those aged ≤80 years, although without significant differences in the CVAE incidence between the SD and RD-TKIs.

Recently, attention has been focused on the dose optimization of TKIs to achieve a delicate balance between efficacy and safety [17]. The feasibility of RD-TKIs has been reported also in elderly patients [4,11,14,26,35,36]. For instance, Seo et al. conducted a comprehensive analysis of TKI dosing patterns in a cohort of 378 patients with a median age of 75 years, revealing that RD-TKIs were administered in 65.9% of patients at the latest follow-up, with no discernible impact on the OS [37]. In our cohort, 36.5% of patients started treatment with RD-TKIs, while the proportion of patients receiving RD-TKIs had increased to >60% in the second line and in all the patients in the subsequent lines. Among them, 27 patients experienced dose reduction due to severe or persistent AEs. In any case, TKIs were well tolerated, and treatment resistance was the main reason for switching the TKIs in each treatment line. Importantly, we observed a significant decrease in grade ≥3 AEs in the first-line RD-TKIs cohort compared to the SD-TKIs (Figure 4b). This reduction in AEs can be primarily attributed to the initial dose reduction strategy.

Furthermore, the comparison between the patients treated with SD-TKIs or RD-TKIs did not show any differences in terms of the OS (Figure 3); on the contrary, the EFS in the standard-dose group was shorter compared to that of the patients in the reduced-dose group, suggesting that in this patient population, the rational use of reduced doses may contribute to improving treatment adherence and maintaining the MMR without compromising the OS.

To date, real-life data on the use of PON in the elderly population are lacking. We reported the outcomes of seven very elderly patients treated with PON at a median age of 82 years (range: 79–84), including two in a second-line setting and five in a third-line setting. In total, the median follow-up was 34 months. All the patients switched treatment due to resistance to previous lines, including two cases with the T315I mutation. Among them, only one patient, who received PON in the third-line setting at a dosage of 15 mg per day, achieved an MMR and experienced DMR within 6 months of treatment. In the remaining patients, the treatment response was influenced by poor adherence to therapy, leading to frequent interruptions and modifications of the treatment schedule. Five patients experienced AEs, including a case of ischemic stroke in a patient with a history of hypertension and dyslipidemia, who was treated with PON 30 mg. Taken together, these data suggest the potential efficacy of PON even in this setting of elderly and heavily pretreated patients. However, caution is required when prescribing PON in very elderly patients, necessitating a careful evaluation of CVR factors and the development of a prevention plan once the medication is initiated. Nonetheless, dedicated studies are necessary to assess the feasibility and true safety of PON in patients aged ≥ 65 years.

We also present the case of a patient of 86 years treated with fourth-line ASC. To date, there are no specific studies available in this setting. Twenty-nine patients aged ≥65 years and four patients aged ≥75 years were included in the pivotal trial ASCEMBL, which compared ASC 40 mg BID with BOS 500 mg QD in patients previously treated with ≥2 TKIs. At the last follow-up at 96 weeks, ASC showed favorable MMR rates in patients aged ≥65 years (4.6%, 95% CI: −25.1 to 34.3) and ≥75 years (50%, 95% CI:−19.3 to 100.0) [38]. Our patient, who was resistant to previous therapies including PON, achieved MMR within 3 months with ASC (200 mg twice daily). Good tolerability was observed, except for a temporary grade 3 elevation in liver transaminases. Further studies are needed to assess the efficacy and safety of ASC, especially in the elderly population.

TFR has become a significant clinical endpoint in many patients with CML [39]. A treatment duration of at least 5 years and a stable MR4 for at least 3 years or 2 years of stable MR4.5 have been identified as optimal prerequisites for discontinuation [18]. Pioneering studies with IMA, followed by those with 2G-TKIs, have demonstrated the feasibility of treatment suspension even in the elderly population [35,40]. Nevertheless, the TFR rate in our cohort was lower than expected, raising several considerations. The patients’ advanced age, exceeding the typical clinical trial inclusion criteria, a median follow-up of 46.62 months, and potential reduced compliance due to age and comorbidities may have contributed to this outcome. Additionally, it is noteworthy that 86.1% of the patients were treated with IMA as a first-line therapy, while only 13.9% received frontline 2G-TKIs, thereby delaying the achievement of the required molecular response for suspension in line with the existing literature. On the other hand, administering reduced doses to five patients did not hinder their ability to achieve optimal molecular responses for discontinuation, indicating that other factors beyond TKIs, such as individual patient characteristics or adherence, could influence the desired molecular responses.

## 5. Conclusions

In conclusion, TKIs have proven to be effective in our cohort of very elderly patients, with 64% of patients achieving an MMR during follow-up. Furthermore, although most of the patients experienced AEs, these were mostly manageable and addressed through dose modifications or specific therapies. Dose reduction strategies, either at baseline or during treatment, represent reasonable approaches in the setting of elderly patients, aiming to achieve an MR, improve treatment adherence, and avoid toxicity, as demonstrated in our analysis. Despite the limitations inherent in retrospective studies, this study provides a real-world representation of “very elderly” patients often excluded from clinical trials. However, further specific studies are needed to evaluate the use of agents such as ponatinib and asciminib in this patient population.

## Figures and Tables

**Table 1:** Patients’ characteristics at diagnosis.

Characteristics	
Patients, *n*	123
M/F, *n* (%)	48/75 (39.0/60.9)
Median age at diagnosis, years (range)	80 (75–96)
SOKAL score, *n* (%)	
Low	1 (0.8)
Intermediate	88 (71.5)
High	34 (27.6)
ELTS, *n* (%)	
Low	4 (3.2)
Intermediate	76 (61.7)
High	43 (34.9)
Comorbidities at diagnosis, *n* (%)	118 (95.9)
Median CCI (range)	1 (0–5)
CCI 0, *n* (%)	55 (44.7)
CCI 1, *n* (%)	28 (22.6)
CCI ≥ 2, *n* (%)	40 (32.5)
Median comorbidities per patient (range)	3.21 (0–9)
Cardiovascular disease, *n* (%)	101 (82.1)
Arterial hypertension	58 (47.1)
Chronic ischemic heart disease	7 (5.6)
Acute myocardial infarction	10 (8.1)
Atrial fibrillation	13 (10.4)
Stroke	6 (4.8)
Transient ischemic attack	7 (5.6)
Chronic obstructive pneumonia disease, *n* (%)	13 (10.5)
Chronic kidney disease, *n* (%)	12 (9.7)
eGFR (CKD-EPI) < 30 mL/min	16 (13.0)
eGFR (CKD-EPI) 30–60 mL/min	38 (30.8)
Dyslipidemia, *n* (%)	43 (34.9)
Diabetes mellitus type 2, *n* (%)	13 (10.4)
Benign prostatic hyperplasia, *n* (%)	20 (16.2)
Cancer history, *n* (%)	22 (17.8)
Breast cancer	6 (4.8)
Colorectal cancer	3 (2.4)
Prostatic cancer	9 (7.1)
Other cancer	11 (8.9)
Patients in treatment for any causes, *n* (%)	116 (94.3)
Median drugs per patient (range)	4.57 (0–13)
Polypharmacy (>5 drugs), *n* (%)	58 (47.1)
Smokers, *n* (%)	14 (11.3)

CCI, Charlson Comorbidity Index; CKD-EPI, Chronic Kidney Disease-Epidemiology Collaboration; ELTS, EUTOS Long-Term Survival; eGFR, estimated Glomerular Filtration Rate.

**Table 2:** Dosages and treatment durations in different lines of therapy.

Line of Treatment	Drug	N. of Patients	Median Dose, mg/day (Range)	Median Duration of Treatment, Months (Range)
First line (*n* = 123)				
	IMA	106 (86.1)	400 (100–800)	41.9 (1–201)
	NIL	7 (5.6)	600 (300–600)	48.2 (7–102)
	DAS	9 (7.1)	100 (50–100)	46.7 (3–102)
	BOS	1 (0.8)	400	31
Second line (*n* = 31)				
	IMA	1 (3.2)	400	77
	NIL	6 (19.3)	600 (400–800)	58.6 (5–116)
	DAS	13 (41.9)	100 (50–300)	46.1 (7–105)
	BOS	9 (29.0)	300 (100–500)	18.4 (3–87)
	PON	2 (6.4)	15–30	39 (8–70)
Third line (*n* = 9)				
	NIL	2 (22.2)	200–400	48 (46–50)
	DAS	1 (11.1)	50	119
	BOS	1 (11.1)	300	21
	PON	5 (55.5)	15 (15–45)	32.2 (1–91)
Fourth line (*n* = 1)				
	ASC	1	400	45

ASC, asciminib; BOS, bosutinib, DAS, dasatinib; IMA, imatinib; NIL, nilotinib; PON, ponatinib.

## Data Availability

The data that support the findings of this study are available from the corresponding author upon reasonable request.

## References

[1] Sokal J.E., Baccarani M., Russo D., Tura S. (1988). Staging and prognosis in chronic myelogenous leukemia. Semin. Hematol..

[2] Hasford J., Pfirrmann M., Hehlmann R., Allan N.C., Baccarani M., Kluin-Nelemans J.C., Alimena G., Steegmann J.L., Ansari H. (1998). A new prognostic score for survival of patients with chronic myeloid leukemia treated with interferon alfa. Writing Committee for the Collaborative CML Prognostic Factors Project Group. J. Natl. Cancer Inst..

[3] Bower H., Björkholm M., Dickman P.W., Höglund M., Lambert P.C., Andersson T.M.L. (2016). Life Expectancy of Patients with Chronic Myeloid Leukemia Approaches the Life Expectancy of the General Population. J. Clin. Oncol..

[4] Breccia M., Tiribelli M., Alimena G. (2012). Tyrosine kinase inhibitors for elderly chronic myeloid leukemia patients: A systematic review of efficacy and safety data. Crit. Rev. Oncol. Hematol..

[5] Maas C.C.H.M., van Klaveren D., Ector G.I.C.G., Posthuma E.F.M., Visser O., Westerweel P.E., Janssen J.J.W.M., Blijlevens N.M.A., Dinmohamed A.G. (2022). The evolution of the loss of life expectancy in patients with chronic myeloid leukaemia: A population-based study in the Netherlands, 1989–2018. Br. J. Haematol..

[6] Pfirrmann M., Clark R.E., Prejzner W., Lauseker M., Baccarani M., Saussele S., Guilhot F., Heibl S., Hehlmann R., Faber E. (2020). The EUTOS long-term survival (ELTS) score is superior to the Sokal score for predicting survival in chronic myeloid leukemia. Leukemia.

[7] Iurlo A., Ubertis A., Artuso S., Bucelli C., Radice T., Zappa M., Cattaneo D., Mari D., Cortelezzi A. (2014). Comorbidities and polypharmacy impact on complete cytogenetic response in chronic myeloid leukaemia elderly patients. Eur. J. Intern. Med..

[8] Helissey C., Biondani P., Roquet F., Lanoy E., Mir O., Varga A., Massard C., Gazzah A., Ribrag V., Bahleda R. (2016). Patients aged over 75 years enrolled in Phase I clinical trials: The Gustave Roussy experience. Int. J. Cancer..

[9] Luskin M.R., DeAngelo D.J. (2018). How to treat chronic myeloid leukemia (CML) in older adults. J. Geriatr. Oncol..

[10] Rohrbacher M., Berger U., Hochhaus A., Metzgeroth G., Adam K., Lahaye T., Saussele S., Müller M.C., Hasford J., Heimpel H. (2009). Clinical trials underestimate the age of chronic myeloid leukemia (CML) patients. Incidence and median age of Ph/BCR-ABL-positive CML and other chronic myeloproliferative disorders in a representative area in Germany. Leukemia.

[11] Latagliata R., Ferrero D., Iurlo A., Cavazzini F., Castagnetti F., Abruzzese E., Fava C., Breccia M., Annunziata M., Stagno F. (2013). Imatinib in very elderly patients with chronic myeloid leukemia in chronic phase: A retrospective study. Drugs Aging.

[12] Senapati J., Sasaki K., Issa G.C., Lipton J.H., Radich J.P., Jabbour E., Kantarjian H.M. (2023). Management of chronic myeloid leukemia in 2023—Common ground and common sense. Blood Cancer J..

[13] Breccia M., Abruzzese E., Castagnetti F., Bonifacio M., Gangemi D., Sorà F., Iurlo A., Luciano L., Gozzini A., Gentile M. (2018). Ponatinib as second-line treatment in chronic phase chronic myeloid leukemia patients in real-life practice. Ann. Hematol..

[14] Latagliata R., Attolico I., Trawinska M.M., Capodanno I., Annunziata M., Elena C., Luciano L., Crugnola M., Bergamaschi M., Bonifacio M. (2021). Bosutinib in the real-life treatment of chronic myeloid leukemia patients aged >65 years resistant/intolerant to previous tyrosine-kinase inhibitors. Hematol. Oncol..

[15] Iurlo A., Galimberti S., Abruzzese E., Annunziata M., Bonifacio M., Latagliata R., Pregno P., Ferrero D., Sorà F., Orlandi E.M. (2018). Pleural effusion and molecular response in dasatinib-treated chronic myeloid leukemia patients in a real-life Italian multicenter series. Ann. Hematol..

[16] Breccia M., Luciano L., Latagliata R., Castagnetti F., Ferrero D., Cavazzini F., Trawinska M.M., Annunziata M., Stagno F., Tiribelli M. (2014). Age influences initial dose and compliance to imatinib in chronic myeloid leukemia elderly patients but concomitant comorbidities appear to influence overall and event-free survival. Leuk. Res..

[17] Iurlo A., Cattaneo D., Bucelli C., Breccia M. (2021). Dose Optimization of Tyrosine Kinase Inhibitors in Chronic Myeloid Leukemia: A New Therapeutic Challenge. J. Clin. Med..

[18] Hochhaus A., Baccarani M., Silver R.T., Schiffer C., Apperley J.F., Cervantes F., Clark R.E., Cortes J.E., Deininger M.W., Guilhot F. (2020). European LeukemiaNet 2020 recommendations for treating chronic myeloid leukemia. Leukemia.

[19] Charlson M.E., Pompei P., Ales K.L., MacKenzie C.R. (1987). A new method of classifying prognostic comorbidity in longitudinal studies: Development and validation. J. Chronic. Dis..

[20] (2017). Common Terminology Criteria for Adverse Events (CTCAE).

[21] Cortes J.E., Gambacorti-Passerini C., Deininger M.W., Mauro M.J., Chuah C., Kim D.-W., Dyagil I., Glushko N., Milojkovic D., le Coutre P. (2018). Bosutinib Versus Imatinib for Newly Diagnosed Chronic Myeloid Leukemia: Results from the Randomized BFORE Trial. J. Clin. Oncol..

[22] Cancer Stat Facts: Leukemia—Chronic Myeloid Leukemia (CML). https://seer.cancer.gov/statfacts/html/cmyl.html.

[23] Schneider J., Algharably E.A.E., Budnick A., Wenzel A., Dräger D., Kreutz R. (2021). High Prevalence of Multimorbidity and Polypharmacy in Elderly Patients with Chronic Pain Receiving Home Care are Associated with Multiple Medication-Related Problems. Front. Pharmacol..

[24] Latagliata R., Stagno F., Annunziata M., Abruzzese E., Iurlo A., Guarini A., Fava C., Gozzini A., Bonifacio M., Sorà F. (2016). Frontline Dasatinib Treatment in a “Real-Life” Cohort of Patients Older than 65 Years with Chronic Myeloid Leukemia. Neoplasia.

[25] Iurlo A., Nobili A., Latagliata R., Bucelli C., Castagnetti F., Breccia M., Abruzzese E., Cattaneo D., Fava C., Ferrero D. (2016). Imatinib and polypharmacy in very old patients with chronic myeloid leukemia: Effects on response rate, toxicity and outcome. Oncotarget.

[26] Latagliata R., Breccia M., Castagnetti F., Stagno F., Luciano L., Gozzini A., Ulisciani S., Cavazzini F., Annunziata M., Sorà F. (2011). Dasatinib is safe and effective in unselected chronic myeloid leukaemia elderly patients resistant/intolerant to imatinib. Leuk. Res..

[27] Cortes J. (2020). How to manage CML patients with comorbidities. Hematology.

[28] Zulbaran-Rojas A., Lin H.K., Shi Q., Williams L.A., George B., Garcia-Manero G., Jabbour E., O’brien S., Ravandi F., Wierda W. (2018). A prospective analysis of symptom burden for patients with chronic myeloid leukemia in chronic phase treated with frontline second- and third-generation tyrosine kinase inhibitors. Cancer Med..

[29] Williams L.A., Gonzalez A.G.G., Ault P., Mendoza T.R., Sailors M.L., Williams J.L., Huang F., Nazha A., Kantarjian H.M., Cleeland C.S. (2013). Measuring the symptom burden associated with the treatment of chronic myeloid leukemia. Blood..

[30] Cortes J.E., Saglio G., Kantarjian H.M., Baccarani M., Mayer J., Boqué C., Shah N.P., Chuah C., Casanova L., Bradley-Garelik B. (2016). Final 5-Year Study Results of DASISION: The Dasatinib versus Imatinib Study in Treatment-Naïve Chronic Myeloid Leukemia Patients Trial. J. Clin. Oncol..

[31] Murai K., Ureshino H., Kumagai T., Tanaka H., Nishiwaki K., Wakita S., Inokuchi K., Fukushima T., Yoshida C., Uoshima N. (2021). Low-dose dasatinib in older patients with chronic myeloid leukaemia in chronic phase (DAVLEC): A single-arm, multicentre, phase 2 trial. Lancet Haematol..

[32] Hughes T.P., Laneuville P., Rousselot P., Snyder D.S., Rea D., Shah N.P., Paar D., Abruzzese E., Hochhaus A., Lipton J.H. (2019). Incidence, outcomes, and risk factors of pleural effusion in patients receiving dasatinib therapy for Philadelphia chromosome-positive leukemia. Haematologica.

[33] Visseren F.L.J., Mach F., Smulders Y.M., Carballo D., Koskinas K.C., Bäck M., Benetos A., Biffi A., Boavida J.-M., Capodanno D. (2021). 2021 ESC Guidelines on cardiovascular disease prevention in clinical practice. Eur. Heart J..

[34] Rodgers J.L., Jones J., Bolleddu S.I., Vanthenapalli S., Rodgers L.E., Shah K., Karia K., Panguluri S.K. (2019). Cardiovascular Risks Associated with Gender and Aging. J. Cardiovasc. Dev. Dis..

[35] Luciano L., Latagliata R., Gugliotta G., Annunziata M., Tiribelli M., Martino B., Sica A., Esposito M.R., Bocchia M., Galimberti S. (2023). Efficacy and safety of nilotinib as frontline treatment in elderly (> 65 years) chronic myeloid leukemia patients outside clinical trials. Ann. Hematol..

[36] Tokuhira M., Kimura Y., Tabayashi T., Watanabe N., Tsuchiya S., Takaku T., Iriyama N., Sato E., Nakazato T., Mitsumori T. (2023). Clinical management of second-generation tyrosine kinase inhibitor therapy in patients with newly diagnosed chronic myeloid leukemia in the chronic phase, focusing on age and dose effects. Int. J. Hematol..

[37] Seo H.Y., Ko T.H., Hyun S.Y., Song H., Lim S.T., Shim K.Y., Lee J.I., Kong J.H. (2019). Tyrosine Kinase Inhibitor Dosing Patterns in Elderly Patients with Chronic Myeloid Leukemia. Clin. Lymphoma Myeloma Leuk..

[38] Hochhaus A., Réa D., Boquimpani C., Minami Y., Cortes J.E., Hughes T.P., Apperley J.F., Lomaia E., Voloshin S., Turkina A. (2023). Asciminib vs bosutinib in chronic-phase chronic myeloid leukemia previously treated with at least two tyrosine kinase inhibitors: Longer-term follow-up of ASCEMBL. Leukemia.

[39] Baccarani M., Abruzzese E., Accurso V., Albano F., Annunziata M., Barulli S., Beltrami G., Bergamaschi M., Binotto G., Bocchia M. (2019). Managing chronic myeloid leukemia for treatment-free remission: A proposal from the GIMEMA CML WP. Blood Adv..

[40] Crugnola M., Castagnetti F., Breccia M., Ferrero D., Trawinska M.M., Abruzzese E., Annunziata M., Stagno F., Tiribelli M., Binotto G. (2019). Outcome of very elderly chronic myeloid leukaemia patients treated with imatinib frontline. Ann. Hematol..

